# Current challenges and opportunities for pharmacogenomics: perspective of the Industry Pharmacogenomics Working Group (I-PWG)

**DOI:** 10.1007/s00439-021-02282-3

**Published:** 2021-06-03

**Authors:** Karina Bienfait, Aparna Chhibber, Jean-Claude Marshall, Martin Armstrong, Charles Cox, Peter M. Shaw, Charles Paulding

**Affiliations:** 1 Present Address: Bristol Myers Squibb, Princeton, NJ 08543 USA; 2 Merck & Co., Inc., Kenilworth, NJ USA; 3 Pfizer, Groton, CT 06340 USA; 4 UCB, Chemin du Foriest 1, 1420 Braine-l’Alleud, Belgium; 5 GSK - Medicines Research Centre, Gunnels Wood Road, Stevenage Hertfordshire, SG1 2NY UK; 6 Regeneron Pharmaceuticals, Inc., Tarrytown, NY 10591 USA

## Abstract

Pharmaceutical companies have increasingly utilized genomic data for the selection of drug targets and the development of precision medicine approaches. Most major pharmaceutical companies routinely collect DNA from clinical trial participants and conduct pharmacogenomic (PGx) studies. However, the implementation of PGx studies during clinical development presents a number of challenges. These challenges include adapting to a constantly changing global regulatory environment, challenges in study design and clinical implementation, and the increasing concerns over patient privacy. Advances in the field of genomics are also providing new opportunities for pharmaceutical companies, including the availability of large genomic databases linked to patient health information, the growing use of polygenic risk scores, and the direct sequencing of clinical trial participants. The Industry Pharmacogenomics Working Group (I-PWG) is an association of pharmaceutical companies actively working in the field of pharmacogenomics. This I-PWG perspective will provide an overview of the steps pharmaceutical companies are taking to address each of these challenges, and the approaches being taken to capitalize on emerging scientific opportunities.

**Supplementary Information:**

The online version contains supplementary material available at 10.1007/s00439-021-02282-3.

## Introduction

The collection of DNA samples from clinical trial participants, especially in early phase clinical trials, has become standard practice for most pharmaceutical companies. However, companies can face a number of challenges when implementing PGx studies, from a shifting global regulatory landscape, to increasing concerns over patient privacy and data access. At the same time, rapid scientific advances in the field of genetics have provided new and emerging opportunities for PGx research. The costs of high-throughput sequencing and genotyping have significantly decreased in the past several years and companies are now routinely performing comprehensive genomic characterization of clinical trial participants. Large databases linking patient health and genomic data have become powerful resources for drug development. These databases can be utilized for the identification of novel targets as well as the deeper characterization of existing targets. The Industry Pharmacogenomics Working Group (I-PWG) is an association of pharmaceutical companies actively working in the field of pharmacogenomics. The I-PWG is comprised of 26 member companies which are collectively running thousands of clinical trials per year with DNA collections as part of the clinical trial protocols. The purpose of this I-PWG perspective is to give an overview of the current challenges and opportunities facing pharmaceutical companies active in the field of clinical pharmacogenomics (PGx). Working to address the challenges outlined in this perspective as well as capitalizing on emerging scientific opportunities will provide researchers and regulators a framework for improving industry sponsored PGx studies for future clinical trials.

### Emerging scientific opportunities: sequencing PGx clinical trial subjects

As the costs of high-throughput sequencing and genotyping have decreased in the past several years, companies are now routinely performing comprehensive genomic characterization of clinical trial participants. An unpublished survey of I-PWG members in 2017 found that 79% of member companies reported using next-generation sequencing (NGS) technologies for internal PGx studies (in at least one study), and more than a third of member companies were widely using these technologies. A total of 53% and 71% of companies reported using NGS for whole-genome and whole-exome sequencing of clinical trial participants, respectively. While NGS technologies were most frequently reported to be used for oncology studies, companies also reported that NGS technologies were being used across a range of other non-oncology therapeutic areas for PGx studies, including cardiovascular, neuroscience, immunology, and rare diseases. The application of NGS to clinical trial samples provides a more comprehensive genomic evaluation of clinical trial participants and may allow for a broader investigation for PGx analysis which includes both common and rare genetic variation (Schwarz et al. 2019).

### Global regulatory challenges for conducting clinical PGx studies

Despite recommendations from regulatory agencies such as the European Medicines Agency (EMA) and Food and Drug Administration (FDA) to collect DNA samples for PGx evaluation across all phases of clinical development (EMA 2018; FDA 2013), global laws and regulations as well as the opinions of individual Investigational Review Boards and Independent Ethics Committees (IRB/IEC) often result in either the inability to collect DNA, or in restrictions on how DNA samples or derived data can be used. The collection and usage of DNA and derived data for PGx research are governed by a complex set of requirements that companies must navigate when conducting global clinical trials. Supplementary Table S1 provides a sampling of laws, regulations and guidances impacting PGx research. This table is not inclusive of all countries and requirements but represents a common subset encountered by sponsors.

Several countries have specific laws aimed explicitly at genetic research or the collection of DNA samples. For example, China’s Regulation of Human Genetic Resources governs the collection, preservation, utilization or provision of China’s human genetic resources to foreign organizations. Recently revised in 2019, this updated regulation strengthened the previously defined oversight activities and added stringent penalties for non-compliance. In practice, multinational companies have experienced requests to provide specific details regarding the assay to be used, the vendor to perform the genetic testing, and the contract with the vendor. These regulations may also require that testing for Chinese participants be performed in China which can lead to greater assay variability for global studies. Taken together, these requirements can hinder the ability for a company to collect and store samples for later use as well as impact the ability for a company to export samples. In addition, the regulation requires any intellectual property (IP) resulting from research to be shared with the Chinese collaborating party (i.e., the clinical site in China). Furthermore, in October of 2019, China passed a new Biosecurity Law which seeks to further intensify the requirements set forth in the regulation of genetic resources. While the impact of this new law is still being assessed by many companies, the collection of biospecimens and assessment of genetics is becoming increasingly challenging, and may serve as an impediment to advancing pharmacogenomic research in China.

In Brazil, both Resolution 340/2004 (NHC 2004) pertaining to genetic research and Resolution 2201/2001 on biorepository and biobank requirements have implications for the conduct of genetic research and storage of genetic specimens, including requirements to share any biobanked samples with investigators in Brazil. In Israel, Guideline for Clinical Trials in Human Subjects (2006) details specific obligations for the conduct of clinical trials with genetic research components, which may include an additional approval step specifically for the genetic research. Finally, many countries have biobanking laws which impact a company’s ability to store, or biobank, genetic specimens for research (e.g., Taiwan (MoHW 2019), Sweden (Regeringskansliet 2002) and Finland (MoSAH 2012)).

Adding to the complexity, other laws/regulations around the globe specifically address the right of an individual to access any genetic information generated from these samples. For example, Brazil’s Resolution 340/2004 establishes the right of a research subject to access their genetic data, to choose whether or not they wish to be informed of results, and for genetic counseling to be provided if requested. Italy’s General Authorization No.8/2014 (IDPA 2014) for the processing of genetic data and Spain’s Biomedical Research Law 14/2007 (Spain 2007) also provide access rights to genetic information. Some countries, including Norway and Argentina (Argentina 2000), have data privacy laws which allow individuals to access personal information collected about themselves, which may include genetic research results, and more broadly, the EU General Data Protection Regulations (GDPR) also provides access rights to personal data (GDPR 2016). Furthermore, some national ethics committees have adopted positions on the return of incidental findings. Denmark’s National Committee on Health Research Ethics (NVK) has adopted guidelines on research that involves comprehensive mapping of personal genomes and requires clinically significant information to be proactively returned to participants if the participant requested to receive such data (NVK 2020). Similarly, Portugal’s Ethics Committee for Clinical Research (CEIC) recently published recommendations for the management of incidental findings from genetic testing in the context of clinical trials.

The complexities involved in the return of genetic information to clinical trial participants have been discussed in detail elsewhere (Downey et al. 2018; MRCT-Center 2017; Prucka et al. 2015). It should be noted that when PGx research is being performed for exploratory purposes, it may be unethical and, depending on the jurisdiction, unlawful to return individual genetic results to participants and their health care providers (Thorogood et al. 2019). PGx research is often performed using research-grade assays that do not meet the level of analytical or clinical validity required for diagnostic testing. For example, in the US, the Clinical Laboratory Improvement Amendments (CLIA) established quality standards for laboratory testing of human specimens for the purposes of diagnosis, prevention, or treatment of disease (CLIA 2003). If genetic research results are not generated in a CLIA-certified lab, and do not meet appropriate analytical standards, these results should not be used as a basis for clinical decision-making, and should be interpreted as exploratory only (MRCT-Center 2017). In addition, the issue of interpretation of results needs to be considered, especially in the context of the generally underpowered exploratory research which is most often performed in the clinical studies. Nonetheless, the return of individual genetic research results remains a challenging issue and sponsors must navigate an uncertain landscape to determine if, when, and how genetic data should be provided back to participants in global trials.

The regional differences in regulations governing conduct of PGx studies, and even the local differences in IRB/EC expectations and preferences, provides challenges for implementing PGx strategies in global studies. Such differences can lead to multiple different restrictions on sample use and data requirements and multiple versions of informed consent documents, which can be challenging to administer and track. While the importance of these regional requirements is understood, the administrative burden to manage them can result in a disincentive to collect and use PGx samples from some regions, meaning that analytical power and applicability of findings across regions may be compromised. Ultimately, we believe there is an opportunity for the scientific and regulatory community to address some of the challenges resulting from this complexity to ensure that important genetic research can be accomplished.

### Challenges for PGx analysis during clinical development

Genetic analyses conducted using clinical trial data can provide an important basis for informed decision-making throughout the clinical development life cycle and can potentially lead to important clinical and commercial opportunities for patient stratification and therapeutic value propositions (Nelson et al. 2016). However, several challenges and limitations exist in the conduct of genetic analyses during clinical development, including limited study size, limited global representation, and difficulties in the validation of findings.

First and foremost, most clinical studies are not designed specifically with a genetic and/or PGx hypothesis as the primary objective. Rather, studies are designed primarily with a therapeutic hypothesis and are powered to detect differences in safety and efficacy, with PGx or genetic objectives as either a tertiary or exploratory objective. Phase I studies are generally underpowered for even candidate variant analyses unless data from multiple studies are pooled (Guo et al. 2019; Kobie et al. 2019). While phase II and phase III studies are larger, even these studies are often underpowered when conducting genome-wide association studies.

A further challenge is the lack of population diversity within clinical trials. The majority of clinical trial participants are of European ancestry (FDA 2017). This imbalance is certainly not unique to genetic analyses conducted in clinical trials and is a well-recognized limitation of the current body of research in genetics more broadly (Popejoy and Fullerton 2016). However, the failure to adequately capture global genetic diversity in PGx studies can result in missed signals that may be important in clinical practice. Indeed, many known clinically relevant PGx biomarkers occur only in or at substantially higher frequencies in non-European populations. For example, HLA-B*15:02, which is associated with cutaneous adverse reactions to carbamazepine or oxcarbazepine, occurs specifically in certain East Asian and South Asian populations (Phillips et al. 2018). In another example, the CYP2C19 poor metabolizer phenotype, which is associated with increased or decreased likelihoods of adverse events or efficacy for a number of different drugs, occurs at much higher frequencies in Asian populations (Scott et al. 2012). Failure to include diverse populations will mean that such associations may not be detected. Further, evaluating the transferability of findings from a genetic association study of drug response conducted in a predominantly European ancestry dataset to other (non-European) populations can be challenging when the number of such subjects available for analysis are limited.

Finally, for new chemical entities and/or drugs with novel mechanisms, the data being generated from early clinical development programs are likely to be the first and only data available, making it difficult to confirm or refute novel genetic findings until additional clinical studies have been conducted. However, differences in clinical trial design, population heterogeneity, and lack of statistical power for replication may make the interpretation of PGx findings from follow-up clinical trials challenging as well (Hopewell et al. 2019; Shen et al. 2020). This uncertainty in interpretation of clinical utility for genetic analyses during drug development is a general disincentive for initiating exploratory analyses because the downside risk of an uninterpretable, unconfirmable exploratory finding may outweigh any upside potential.

### Challenges in PGx ADME studies

Genetic variants that alter activity of drug metabolizing enzymes and drug transporters are responsible for many known PGx associations (FDA 2015; Tremaine et al. 2015). By altering enzyme or transporter activity, such variants can drive inter-individual variability in exposure, and potentially have an impact on drug safety or efficacy if the variability in exposure exceeds the therapeutic window for small molecules. In addition to issues with limited clinical trial size discussed previously, there are several challenges unique to the study of PGx in early phase studies. These include the potential uncertainty in determining metabolism pathways for new compounds in early clinical development, and the continued emergence of variants with clinical significance.

Ideally, PGx analyses in early phase studies would be conducted in a targeted fashion, prioritizing variants in genes that have been shown via preclinical work to be important for the disposition of the compound to maximize likelihood of success in conducting analyses in very small trial datasets. However, preclinical in vitro studies of the major and minor pathways of metabolism are often not completed before the initiation of phase II or even pivotal studies. For this reason, it is often important to pool as many early phase clinical studies with pharmacokinetic (PK) data as possible to boost statistical power to assess a broader set of ADME genes. In addition, larger phase II/III studies can also be used where estimates of PK parameters can be derived using population PK modeling to assess potential impact of variants in ADME genes (Guo et al. 2019; Kobie et al. 2019). Even so, the statistical power to detect genetic associations in these data sets may not be sufficient. In particular, power to detect rare variants that could impact safety exposure is almost always limited, especially for patients who may have more than one functional mutation in a set of metabolizing enzymes. Rational drug design over the past several decades has mostly eliminated drugs that are predominantly metabolized by highly polymorphic Cytochrome P450 enzymes (CYPs) such as CYP2D6, in favor of spreading the fraction metabolized across several CYPs or other enzyme families where possible. That does not fully eliminate the chance of a patient having a poor metabolizer phenotype in two of those enzymes which may lead to variability in exposure that could be clinically meaningful. Furthermore, other metabolic clearance routes such as glucuronidation and the role of membrane transporters have emerged as being potentially relevant for PGx studies (Desai et al. 2003; Guillemette 2003; Yee et al. 2018). While there is generally less strong prior clinical evidence to support the functional impact of genetic variants in these other classes of metabolic enzymes or in membrane transporters, PGx analyses will likely be required to understand their potential impact on PK and pharmacodynamics.

It is therefore recommended to carefully assess the metabolism pathways of all upcoming clinical candidates and to conduct genotyping for ADME genes in early and late phase clinical trials. If a drug is metabolized by pathways with known polymorphic variation, for which there is strong evidence supporting clinically meaningful effects for other approved drugs, then studying the potential impact is likely warranted during development of the new chemical entity. In addition, the EMA has released PGx guidelines that recommend the potential use of broader, whole-exome or whole-genome sequencing to assess potential novel variants in cases where unexpected PK variability exists that is not explainable by traditional PGx genotyping (EMA 2018). Broader genome-scale sequencing comes with additional challenges, including the need for phenotypic confirmation of novel variants, which may be required by regulatory agencies.

### Challenges of clinical implementation

Despite the investment of resources by industry and academia in identifying PGx biomarkers, and a growing list of clinically relevant markers that could be used to improve patient care (FDA 2015; Relling et al. 2020), such information is still not widely used in clinical practice. The reasons for this are diverse and have been reviewed in depth elsewhere (Chenoweth et al. 2020; Klein et al. 2017); examples include difficulties in the ordering, reimbursement and interpretation of genetic tests, the lack of education for both patients and clinicians, and limited evidence supporting the clinical utility and health economic value of many PGx biomarkers. In addition, differences in recommendations for PGx testing included in drug labels across regulatory agencies for the same drug have been identified (Koutsilieri et al. 2020; Shekhani et al. 2020); the lack of consensus guidelines for genetic testing and implementation may be an additional hurdle for clinicians attempting to incorporate PGx information in clinical practice.

Efforts have been made to address some of these roadblocks to clinical implementation. Groups like the Clinical Pharmacogenetics Implementation Consortium (CPIC) and Dutch Pharmacogenomics Working Group (DWPG) provide guidelines for the use of PGx information in clinical practice (Bank et al. 2018). In an effort to make test results readily available, pre-emptive PGx testing has been implemented in certain medical centers and health systems (Cecchin et al. 2017; Dunnenberger et al. 2015), eliminating the need for a physician to order a test before prescribing a medication. Studies attempting to demonstrate the clinical validity, utility, and economic value of PGx biomarkers have been conducted for certain widely used drugs (Anderson et al. 2007; Claassens et al. 2019; Pereira et al. 2020; Wadelius et al. 2009; Zhu et al. 2020). However, the use of genetic tests in clinical practice remains limited and the need for a PGx companion diagnostic is generally viewed as a significant hurdle in the development and marketing of new drugs. As the use of PGx information in clinical practice slowly becomes more widespread, it is likely that the importance of and investment in PGx research by industry will continue to grow.

It is also worth noting that, in many cases, drug response is likely to be highly complex, resulting from the interaction of many influencing factors including environmental, anthropometric and genetic factors, as well as biological subsystems affected by the disease (Armstrong 2008). In this respect, it is unlikely that any single genetic marker, biomarker or other single stratifying factor will fully capture this complexity and have appropriate predictive performance for clinical utility, thus limiting test uptake. This is leading to the exploration of emerging opportunities in this field, such polygenic risk scores (see following section) and machine learning approaches. Advances in machine learning approaches, allied with increases in computational power, allow the integration of diverse data types which, when considered together, contribute to determining drug response, improving predictability and supporting translation to clinical practice.

### Emerging scientific opportunities: using polygenic risk scores in PGx studies

An area of emerging scientific interest for PGx studies is the use of polygenic risk scores. Several studies in coronary artery disease (CAD) have suggested that polygenic risk scores may be useful for precision medicine approaches. Two retrospective studies reported that patients with high CAD polygenic risk scores show greater clinical benefit with statin treatment across several clinical trials (Mega et al. 2015; Natarajan et al. 2017). Similarly, two large independent retrospective studies showed that patients with high CAD polygenic risk scores also have greater clinical benefit from treatment with PCSK9 inhibitors in two large outcome trials (Damask et al. 2020; Levin and Rader 2020; Marston et al. 2020). Polygenic risk scores have also been studied in the prevention of atherothrombotic events. In a retrospective PGx study of clopidogrel, Lewis et al. identified a polygenic risk score that was associated with increased platelet reactivity, risk of developing major adverse cardiovascular events, and risk of cardiovascular death (Lewis et al. 2020). Finally, in the field of oncology a recent study found that high vitiligo, high psoriasis, and low atopic dermatitis polygenic risk scores were associated with longer overall survival after treatment with atezolizumab (anti-PD-L1) monotherapy compared to treatment with chemotherapy in bladder cancer patients (Khan et al. 2020).

Polygenic risk scores may also be used to enrich patients for clinical trial enrollment. Patients with high polygenic risk scores can have levels of disease risk similar to those observed in patients with monogenic diseases (Khera et al. 2018). Selectively enrolling patients with high polygenic risk scores may reduce the size of the clinical trials or, for event driven studies, shorten the duration of the study. While the relationship between polygenic risk scores and drug response is an emerging question in drug development, going forward this will likely be an area of scientific interest across a range of diseases and therapeutic areas, and has the potential to have an impact on clinical trial design.

The regulatory implications of applying approaches such as polygenic risk scores and machine learning to patient selection strategies in clinical studies need to be considered, specifically as to how they will impact drug labeling and any companion diagnostic requirements. Regulatory authorities are interested in the ability to target treatments to patients who would most benefit, needing to ensure that the label accurately reflects the enrichment strategies used to select the patients, and that there is an approved method available to identify these patients once any drug is approved. In general, the potential effect of enrichment strategies on labeling and the route of approval of any test as a companion diagnostic should be part of an ongoing dialogue with regulatory bodies during drug development (FDA 2019).

### Emerging scientific opportunities: large genomic databases for drug development

Pharma companies are increasingly utilizing, through either partnerships, collaborations, or acquisitions, large genomic datasets linked to patient health and medical data. Some examples of these consortiums or partnerships are shown in Table 1. These databases provide a valuable resource for broad-based genomic studies relevant for drug development. The major focus of these research efforts is to identify disease associated genes and novel drug targets (Dewey et al. 2016; Szustakowski et al. 2020; Van Hout et al. 2020). However, these large-scale genomic databases can provide an unprecedented level of information for both the safety and efficacy of current clinical development programs and already marketed drugs (Diogo et al. 2018; McInnes et al. 2020). Drug targets with supporting genetic evidence have been shown to have greater success rates in drug development (Nelson et al. 2015). Different classes of genetic variation can provide valuable information for drug targets. In particular, loss of function (LOF) variants have received a great deal of interest for drug development. This class of genetic variation when found to be protective against disease risk can mimic the effects of therapeutic antagonists (e.g. PCSK9) (Cohen et al. 2006). The characterization of drug targets via phenome-wide association studies (PheWAS) within these large databases can also identify novel indications, related indications or even potential safety signals (Diogo et al. 2018; Jerome et al. 2020). Furthermore, the information gleaned from these large databases can also provide additional supportive data for variants identified from PGx analysis of ongoing clinical trials for drugs still in development. Finally, distinct genetic patient subpopulations may be identified for targeted precision medicine clinical development programs, or for call back studies for deeper patient phenotyping.

**Table 1:** Pharmaceutical company partnered genomic health databases

Pharma companies	Partner/consortium	Cohort	Year
Amgen	Decode genetics^1^	160,000 Icelandic participants linked to medical and health records	2012
Regeneron	Geisinger health system	> 100,000 exome-sequenced and array-genotyped subjects linked to electronic health records (EHR)	2014
Abbvie, Alnylam, AstraZeneca, Bristol-Myers Squibb, Biogen, Pfizer, Regeneron, and Takeda	UK Biobank	500,000 UK subjects exome-sequenced and array-genotyped with health and medical records	2018
AbbVie, AstraZeneca, Biogen, Celgene/Bristol-Myers Squibb, Genentech (a member of the Roche Group), GSK, Janssen, Maze Therapeutics, MSD, Novartis, Pfizer and Sanofi	FinnGen	269,000 Finns with combined genotype and health registry data	2017
AbbVie, Alexion Pharmaceuticals, AstraZeneca, Biogen, Dimension Therapeutics, GSK, Helomics^2^, Roche, Takeda, UCB	GENE consortium, Genomics England	Sequence data from 100,000 whole genomes from NHS patients with rare diseases and their families, as well as patients with common cancers	2015

^1^Decode was acquired by Amgen

^2^Helomics is an AI-based data analytics company

### Summary

Going forward pharmaceutical companies will continue to invest heavily in genomic technologies, databases, and PGx studies. Genomics has become an integral part of drug development, from early target discovery through the late stages of clinical development. The challenges outlined in this perspective, while significant, will continue to be addressed going forward as pharmaceutical companies pursue precision medicine strategies broadly across drug development. In particular, the constantly changing legal and regulatory environment for conducting global studies may be the most challenging to overcome. While concerns over patient privacy and the misuse of patient data are valid concerns, overly restrictive policies will impede the advancement of PGx discoveries and will ultimately impede advances in precision medicine more broadly to global populations. More optimistically, advances in genomic technologies are progressing at a rapid pace and pharmaceutical companies are embracing these advances. The incorporation of patient level sequencing, polygenic risk scores, and data from large EHR/genomic databases will likely help seed the discoveries of clinical trial PGx studies in the coming years.

## Supplementary Information

Below is the link to the electronic supplementary material.

Supplementary file1 (DOCX 32 KB)
