# Supplementary material for: Current challenges and opportunities for pharmacogenomics: perspective of the Industry Pharmacogenomics Working Group (I-PWG)
Source: Hum Genet. 2021 Jun 3;141(6):1165–73. doi: 10.1007/s00439-021-02282-3 (PMC9177658; doi:10.1007/s00439-021-02282-3)
Supplement: Supplementary file 1 — Supplementary file1 (DOCX 32 KB) [file 439_2021_2282_MOESM1_ESM.docx]

**Table S1. Sample of global laws, regulations, guidelines impacting pharmagenomic research in global clinical trials.**

| **REGION/**  **COUNTRY** | **REQUIREMENT** | **TYPE** |
| --- | --- | --- |
| European Union | Regulation (EU) 2016/679 of the European Parliament and of the Council of 27 April 2016 on the Protection of Natural Persons with Regard to the Processing of Personal Data and on the Free Movement of Such Data, and Repealing Directive 95/46/EC (General Data Protection Regulation) (GDPR)  *Countries listed below with an asterisk are part of the European Union where GDPR applies  European Medicines Agency (EMA) Guideline on Good Pharmacogenomic Practice (2018) | Legislation  Guideline |
| Argentina | Personal Data Protection Act No. 25.326 (2000) | Legislation |
| Australia | Privacy Act 1988 (2019)  National Health and Medical Research Council (NHMRC) Statement on Ethical Conduct in Human Research (2018) | Legislation  Guideline |
| Belgium* | Belgian Advisory Committee on Bioethics Opinion No. 45: Human Biological Material Banks Intended for Research (2009) | Guideline |
| Brazil | General Data Protection Law (2018)  Ordinance No. 2.201/2011: Establishing the National Guidelines for Biobanks of Human Biological Material for Research Purposes  National Commission on Research Ethics (CONEP) Resolution CNS No. 441/2011: Storage of Human Biological Material or Use of Material Stored in Previous Research  CONEP Decree CNS No. 2201/2001: The National BioRepository and Biobank Guideline  CONEP Resolution CNS No. 340/2004 Research on Human Genetics | Legislation  Legislation  Regulation  Regulation  Regulation |
| Canada | Tri-Council Policy Statement - Ethical Conduct for Research Involving Humans (2018) | Guideline |
| China | People’s Republic of China (PRC) Human Genetic Resources Management Regulations (2019)  PRC Biosecurity Law (2020) | Regulation  Legislation |
| Colombia | Scientific, Technical, and Administrative Regulations for Health Research, Resolution No. 008430, Title III, Chapter II (1993) | Regulation |
| Denmark* | National Committee on Health Research Ethics (NVK) Guidelines on the Use of Biological Material in Health Research Projects (2017)  NVK Guidelines on Health Research Projects Involving Genome Research (2018) | Guideline  Guideline |
| Finland* | Act on the Medical Use of Human Organs, Tissues, and Cells No. 101/2001  Law on Biobanks, No 688/2012  Decree on Consent for Biobank No. 643/2013  Decree on Information on Biobank No. 649/2013  Medical Research Act No. 488/1999 (Amended 295/2004, 794/2010, and 143/2015) | Legislation  Legislation  Regulation  Regulation  Legislation |
| France* | National Commission of Information and Liberty (CNIL): Decree N° 2019-536 of 29 May 2019 Enacted for the Application of Act No. 78-17 of 6 January 1978 on Data Processing, Data Files, and Civil Liberties  CNIL Health Research with Consent (2018)  CNIL Health Research without Consent (2018)  CNIL Practical Guide on the Protection of Personal Data: What Framework Applies to Research? (2018)  National Consultative Bioethics Committee for Health and Life Sciences (CCNE): Ethical Reflection on Developments in Genetic Testing in Connection with Very High Throughput Human DNA Sequencing (2016) | Regulation  Guideline  Guideline  Guideline  Guideline |
| Germany* | Federal Data Protection Act (BDSG) (2017)  German Ethics Council Opinion on Human Biobanks for Research (2010)  German Society of Human Genetics: DNA Banking and Personal Data in Biomedical Research: Technical, Social and Ethical Questions (2004) | Legislation  Guideline  Guideline |
| Hungary* | Act XXI of 2008 on the Rules of Protection of Human Genetic Data, of Human Genetic Examinations and Research and of the Operation of Biobanks | Legislation |
| Ireland* | Health Products and Regulatory Authority (HPRA): Human Biological Material: Recommendations for Collection, Use, and Storage in Research (2005)  HPRA: Guidelines for Pharmacogenetic Research (2006) | Legislation  Guideline  Guideline |
| Israel | Genetic Information Law (2000)  Ministry of Health Guidelines for Clinical Trials in Human Subjects (2006) | Legislation  Guideline |
| Italy* | General Authorisation No. 8/2014 for the Processing of Genetic Data  Provision 146/2019 for the processing of special categories of data in compliance with the GDPR | Legislation  Legislation |
| Japan | Ministry of Health, Labor and Welfare (MHLW) Ethics Guidelines for Human Genome/Gene Analysis Research (2017) | Guideline |
| Netherlands* | Medical Research Involving Human Subjects Act (2006)  Federation of Biomedical Scientific Societies Code for Proper Secondary Use of Tissue in the Netherlands (2002) | Legislation  Guideline |
| New Zealand | Human Tissue Act (2008)  National Ethics Advisory Committee National Ethical Standards: Health and Disability research and Quality Improvement (2019) | Legislation  Guideline |
| Norway* | Act Relating to the Application of Biotechnology in Human Medicine, etc. (Biotechnology Act) (2003)  Act on Health Care Research (2008)  National Committee for Medical and Health Research Ethics (NEM) Guidelines for Genetic Research in Humans | Legislation  Legislation  Guideline |
| Portugal* | Genetic Information Law 12/2005  Ethics Committee for Clinical Research recommendation for the management of “Incidental Findings” in the context of Clinical Research and specifically Clinical Trials | Legislation  Guideline |
| Singapore | Human Biomedical Research Act (2015) | Legislation |
| South Africa | National Health Act No. 61 (2003)  Regulations Relating to the Use of Human Biological Material (2012)  Medical Research Council of South Africa (MRC) Guidelines on Ethics in Reproductive Biology and Genetic Research (2002) | Legislation  Regulation  Guideline |
| South Korea | Ministry of Health and Welfare (MOHW) Bioethics and Safety Act no. 15188 (2017) | Legislation |
| Spain* | Biomedical Research Law 14/2007  Royal Decree 65/2006 of Requirements for the Import and Export of Biological Samples | Legislation  Regulation |
| Sweden* | Biobanks in Medical Care Act No. 297 (2002)  Act on Genetic Integrity (2006) | Legislation  Legislation |
| Taiwan | Human Biobank Management Act (2012)  Regulations on Human Trials (2009):  Regulations on Commercial Benefit Feedback of Human Biobanks (2010)  Administrative Regulations on the Establishment of Human Biobanks (2011)  Ministry of Health and Welfare (MOHW) Guidelines for the Collection and Use of Human Specimens for Research (2006) | Legislation  Regulation  Regulation  Regulation  Guideline |
| United Kingdom | Data Protection Act (2018)  Human Tissue Act (2004) | Legislation  Legislation |
| United States | Protection of Human Subjects (Common Rule), 45 CFR Part 46 (2018)  Health Insurance Portability and Accountability Act (HIPAA)* (1996) (Note sponsors are generally not covered entities under HIPAA, however, clinical trial sites are).  Food and Drug Administration (FDA) Guidance for Industry on Clinical Pharmacogenomics: Premarket Evaluation in Early-Phase Clinical Studies and Recommendations for Labeling (2013)  FDA Guidance on Informed Consent for In Vitro Diagnostic Device Studies Using Leftover Human Specimens That are Not Individually Identifiable (2006)  Office of Human Research Protections (OHRP) Guidance on the Genetic Information Nondiscrimination Act: Implications for Investigators and Institutional Review Board (2009) | Regulation  Regulation  Guideline  Guideline  Guideline |

Note: For a more complete overview of all global requirements related to the conduct of research, please see International Compilation of Human Research Standards (2020) compiled by the Office for Human Research Protections from the U.S. Department of Health and Human Services.
